# Surgery

**Published:** 1877-04

**Authors:** 


### III Surgery.

*Necrosed Nasal Bones Swallowed and Impacted in the Œsophagus.* Dr. M. Langenbeck. (*Memorabilien*, XXII., 1.)

A woman, aged forty years, who four years since had syphilitic caries of the nasal bones, one morning woke up and could not swallow. Though the patient was not aware of having swallowed a hard substance, the œsophagus probe was arrested in its progress, midway between the pharynx and stomach, by a hard body so firmly impacted, that it baffled all attempts at extraction, by various instruments. The patient unable to swallow water, was fed by enemas; but after twenty days it became evident that she would soon succumb from inanition. The doctor then made another attempt at extraction; he introduced a long, slender whalebone, which tapering off toward the end, carried a small, conical bulb. After manipulating about half an hour, the bulb slipped down below the foreign body, and the latter finally began to move when cautious tractions had repeatedly been made with the bulbous end of the whalebone. No sooner was the foreign body dislodged, than it was thrown up by vomiting. It proved to be a conglomerate of the necrosed nasal bone, vomer and both the inferior turbinated bones which the patient must have swallowed in her sleep.

*Pulsating Tumor of Orbit resembling True Aneurism; Ligation of Common Carotid; Subsequent Removal of Tumor; Recovery.* Frothingham. (*The American Jour. of the Med. Sciences*, January, 1877.)

A pulsating tumor of orbit, of three years' standing, occurred in a lady aged thirty-five years. On tactile examination the eye moved perceptibly with each pulsation; and upon compressing the common carotid the bruit ceased, and the tumor, with slight pressure with the finger, would recede in the socket. The eye was very much protruded, and the sight greatly impaired. Later in the month (March, 1872,) Prof. T. A. McGraw saw the case with Dr. F., and diagnosed true aneurism of the orbit. In May—three months later—the common carotid was tied, tumor diminished in size, bruit and pulsation ceased for fourteen days, when it returned, but not with so much force. On the eighteenth day the ligature came away; patient making a slow recovery from the effect of operation. Ligating the artery so greatly retarded the growth of the tumor that not until November 3, 1875, did it become urgent for a second operation.

Diagnosis having been changed from a true aneurism of orbit to aneurism by anastomosis, excision of tumor was advised and made. The excised mass consisted of sacculated vessels, held together by connective tissue, and resembling in appearance a sponge.

Dr. F. believes that there is a general tendency on the part of surgeons to regard all deep-seated pulsating tumors of orbit true aneurisms; and not until a more complete diagnostic distinction is arrived at can mistakes be avoided. W. F. L.

*Method of Retention of Umbilical Hernia at an Early Age.* ARCHAMBAULT. (*Jour. des Conn. Méd-Chir.*, September 1, 1876.)

Very young infants affected with umbilical hernia are generally dressed with a button pad and bandage, held *in situ* by a circular spring. This has the obvious disadvantage that the button rarely remains in coaptation with the umbilical depression, and the consequence is that the remedy becomes worse than the evil.

A simpler method, requiring no bandaging, has been devised by the author. A piece of softened white wax is moulded between the fingers until it has the shape and size of a small ball. It is then divided so as to form two hemispheres. One of these hemispheres, in size proportioned to the extent of the umbilical depression, is engaged by its spherical surface in the depression, and retained in position by a strip of diachylon plaster. Instead of the wax, a bit of gutta-percha may be employed, previously softened in warm water.

Whichever substance is employed the effect produced is the same; at the end of two hours the obturating body is sufficiently softened to adhere firmly to the skin. After that no bandage is necessary to hold it in place. By this simple and inexpensive expedient the cure is effected in less than two months.

If the plaster occasions a cutaneous erythema, it may be replaced every second day, after the surface has been well dusted with rice powder.

*The Gunshot Wounds of the Ankle Joint during the Franco German War.* GROSSHEIM. (*Deutsche Militaeraerztl. Zeitschr.*, 1876, No. 4; *Centralbl. f. Chir.*)

The author could give a pretty complete report of those cases which were submitted to operations, while the record of cases treated by the conservative method is very deficient.

*The total excision of the ankle joint* (*i. e.*, the removal of the articular ends of the tibia, fibula and astragalus,) was recorded fifty times, with twenty-six recoveries, twenty deaths, and four unknown results. In nine cases the leg had to be amputated afterward; two of these patients recovered and seven died. The result of the successful operations was a complete anchylosis, with a shortening of the leg varying between two and fifteen centimetres. In four cases the foot could not be used in walking.

*The partial excision* (*i. e.*, the removal of the articular end of either the tibia or the fibula or the astragalus.) was executed in forty-seven cases, with thirty-three recoveries and fourteen deaths. Secondary amputation became necessary in two cases, one of which died. The shortening varied from 2 to 10.5 centimetres. The functional result in the thirty-two cases: three patients had good, active motion in the joint, and could walk well with the foot; twenty-five patients recovered with anchylosis, but had a more or less good use of their foot, while three patients could not use the foot at all.

*Pirogoff's operation* was recorded twenty-nine times, with thirteen deaths; *Syme's operation* was performed fourteen times, with five deaths; *amputation of the leg* was performed one hundred and forty-five times, with sixty-one deaths.

*Treatment of Gunshot Wounds of the Elbow Joint in the Franco-German War.* DOMINIK. (*Deutsche Militaeraerztl. Zeitschrift*, 1876; *Centralbl. f. Chir.*)

For this paper the author has collected from the official lists all the wounds of the elbow joint treated in the military hospitals established during the last war. He also gathered from the medical journals all those cases of excision of the elbow which were treated in other quarters during the war, and consequently were not contained in the official lists. By this labor the author believes he has obtained nearly a complete list of all the operations performed on the elbow joint by German surgeons in the German hospitals. As to the ultimate result of the treatment, the author's information is mainly drawn from the examinations of the invalids at the pension office during the years 1871 to 1873. In regard to French soldiers, he gathered some valuable information from Chenu "Apercu historique statisque et clinique sur le service des ambulances et des hôpitaux pendant la guerre de 1870–1871." In this way the author succeeded in obtaining information of the ultimate result in 263 cases of excision of the elbow.

1. The *conservative treatment* of the gunshot wounds of the elbow during the wars from 1848 to 1866 shows an aggregate mortality of 46.8 per cent., the excision 21.1 per cent., and the amputation 33.3 per cent. During the last war the results of the conservative treatment were far better because the lighter lesions only were put under this treatment, and whenever any untoward symptoms appeared, secondary excision or amputation were performed. For these reasons but fifty-one cases could be collected which were conservatively treated to the last, of which 5=9.8 per cent. died.

In a table, the writer classifies the functional results of 163 cases of gunshots of the elbow, treated conservately during modern wars : Free mobility in 10 cases (mostly flesh wounds, without lesion of the bones); incomplete anchylosis in 18 cases; complete anchylosis in 133 cases; result unknown in 2 cases.

The 133 cases of complete anchylosis were divided in: 1, hand and fingers could be used in 12 cases; 2, hand and fingers were paralyzed in 49 cases; 3, functions not stated in 72 cases. From all this, the author concluded that the conservative method, though generally applied to the milder lesions, did not yield a favorable result. It almost invariably terminated with anchylosis.

2. The *excision of the elbow* for gunshot wounds was performed in 400 cases, with 90 deaths; pyemia and septicemia, 68; trismus, 1; diphtheria, 3; gangrene, 1; erysipelas, 1; pneumonia, 3; exhaustion, 2.

The ultimate result noted in 263 cases, was as follows:

Good, active motion of elbow joint, in 28 cases;

Limited motion of elbow joint, good use of hand, in 35 cases;

Anchylosis (without further data), in 43 cases;

Anchylosis, good use of the hand, in 55 cases;

Anchylosis, paralyzed hand, in 31 cases;

Incomplete anchylosis, paralysis of hand, in 6 cases;

Loose joint, useful hand, in 24 cases;

Loose joint, no use of hand, in 41 cases;

As to the bone excised, the statistics of the writer tend to prove that the best functional results are obtained by the excision of the epiphyses of the fore-arm, while the epiphysis of the humerus is preserved. The excision of one bone of the fore-arm is most frequently followed by anchylosis, while the excision of the end of the humerus is the most frequent cause of a dangling arm.

3. *Amputation of the arm* for gunshot wound of the elbow joint. The writer compiled 137 cases of this operation, with 48 deaths, and 167 cases of the same operation for other injuries, with 53 deaths. The mortality of the amputations performed on the first day, was 22 per cent.; on the second day, 55 per cent.; from third to sixth day, 26 per cent.; and later than the first week, 39 per cent.

